# AOA-Based Three-Dimensional Multi-Target Localization in Industrial WSNs for LOS Conditions

**DOI:** 10.3390/s18082727

**Published:** 2018-08-19

**Authors:** Ruonan Zhang, Jiawei Liu, Xiaojiang Du, Bin Li, Mohsen Guizani

**Affiliations:** 1 Department of Communication Engineering, Northwestern Polytechnical University, Xi’an 710072, China; rzhang@nwpu.edu.cn (R.Z.); ljw0823@mail.nwpu.edu.cn (J.L.); libin@nwpu.edu.cn (B.L.); 2 Department of Computer and Information Sciences, Temple University, Philadelphia, PA 19122, USA; 3 Department of Electrical and Computer Engineering, University of Idaho, Moscow, ID 83844, USA; mguizani@ieee.org

**Keywords:** wireless sensor network, localization, smart industrial, angle of arrival, maximum likelihood estimator, antenna array

## Abstract

High-precision and fast relative positioning of a large number of mobile sensor nodes (MSNs) is crucial for smart industrial wireless sensor networks (SIWSNs). However, positioning multiple targets simultaneously in three-dimensional (3D) space has been less explored. In this paper, we propose a new approach, called Angle-of-Arrival (AOA) based Three-dimensional Multi-target Localization (ATML). The approach utilizes two anchor nodes (ANs) with antenna arrays to receive the spread spectrum signals broadcast by MSNs. We design a multi-target single-input-multiple-output (MT-SIMO) signal transmission scheme and a simple iterative maximum likelihood estimator (MLE) to estimate the 2D AOAs of multiple MSNs simultaneously. We further adopt the skew line theorem of 3D geometry to mitigate the AOA estimation errors in determining locations. We have conducted extensive simulations and also developed a testbed of the proposed ATML. The numerical and field experiment results have verified that the proposed ATML can locate multiple MSNs simultaneously with high accuracy and efficiency by exploiting the spread spectrum gain and antenna array gain. The ATML scheme does not require extra hardware or synchronization among nodes, and has good capability in mitigating interference and multipath effect in complicated industrial environments.

## 1. Introduction

The wireless sensor network (WSN) is a highly integrated discipline and has been well developed in the last decade [1]. WSNs have enabled smart industry by exploiting the significant advantages such as extremely low power consumption [2], self-organization, and on-site sensing. In typical smart industrial WSNs (SIWSNs), plenty of sensor nodes are deployed in industrial facilities to monitor manufacturing processes, compute simple tasks, collect and deliver information [3]. SIWSNs can support a broad range of applications, such as factory automation, equipment management, fault diagnosis, surveillance, etc. However, SIWSNs also pose challenges due to the stringent requirements on reliability, latency, and security [4,5], and their susceptibility to the highly complicated environments with serious electromagnetic interference and jamming [6]. Hence, we need to enhance the functionality and reliability of conventional WSNs to satisfy the requirements of industrial-grade applications.

One important issue for SIWSNs is the node localization. Wireless sensors/actuators are usually placed at specified moving points in industrial environments. For example, mobile sensor nodes (MSNs) may be mounted on the carrying facilities in wharves, freight terminals, and mines. It is critical to locate the MSNs quickly and accurately for target tracking and process monitoring. The global positioning system (GPS) is not applicable because SIWSNs usually operate in indoor or underground environments without the GPS signals. Therefore, the efficient, precise, and fast determination of relative positions of multiple MSNs is an important and challenging issue for SIWSNs.

Although wireless positioning methods have been studied extensively (as reviewed in Section 2), the current schemes most focus on positioning in two-dimensional (2D) planes. The positioning in three-dimensional (3D) space has been less explored. Another key limitation is that the current methods usually locate a single node/target in one measurement trial and cannot perform multi-target positioning simultaneously. As mentioned earlier, to locate a number of MSNs quickly is an essential feature for industrial applications.

Motivated by the characteristics of SIWSNs and the deficiencies of current positioning algorithms, this paper proposes a new scheme, called Angle-of-arrival (AOA) based three-dimensional Multi-target Localization (ATML). This algorithm can provide fast simultaneous localization of multiple MSNs with small operational latency. The main contributions of this paper are as follows.

First, the ATML algorithm based on the iterative maximum-likelihood (ML) method is proposed. We design the sensor network architecture that has two anchor nodes (ANs) with fixed positions and a number of MSNs whose positions relative to the ANs are to be determined. We propose a multi-target single-input-multiple-output (MT-SIMO) signal transmission scheme that combines the code division multiplexing access (CDMA) and ML estimation. Each MSN is assigned a pre-defined orthogonal spread spectrum sequence and broadcasts the spreading sequence using an omnidirectional antenna. The ANs capture the signals from all the MSNs simultaneously using antenna arrays. Then, we adopt the interactive ML method to estimate the 2D AOAs of the line-of-sight (LOS) paths from each MSN to the ANs in the joint time-space domain. Finally, the relative positions of the MSNs in the 3D space are deduced according to the 3D geometric theory of skew lines.

Second, we conduct extensive simulations to evaluate and compare the performance of ATML with other methods in the literature. The simulations of AOA estimation and localization errors for varying the signal-to-noise ratio (SNR), number of MSNs, and AN antenna array size are presented. The results show that ATML can locate multiple MSNs simultaneously with high accuracy.

Third, we have developed a testbed of ATML and performed field experiments to validate the scheme. The prototype system comprises two portable transmitters (Tx) that send orthogonal pseudo-noise (PN) sequences simultaneously to emulate MSNs and a receiver (Rx) that captures the signals using a rectangular planar antenna array to emulate an AN. The antenna array comprises 64 elements and the system operates at the carrier frequency of 3.5 GHz. In the experiment, the two Tx’s were moved along a line, and the AOAs of their LOS paths to the AN were correctly determined. The field experiment has verified the effectiveness and feasibility of ATML in practical systems.

The proposed ATML provides a fast multi-target localization method suitable for SIWSNs with movability, low transmission power, and low cost. It has the following advantages.

- The designed MT-SIMO signal transmission scheme utilizes the existing communication systems without the need of extra cost, weight, and volume for new hardware. The scheme requires high hardware complexity and computational capacity on ANs, and minimizes the complexity and working load of MSNs, which is preferable in WSNs.
- ATML adopts a simple iterative ML method to estimate the azimuth and elevation AOAs of propagation paths, which enables the 3D localization. The simulation and experimental results demonstrate the high accuracy of the 2D AOA estimation.
- By using the CDMA approach, the spread spectrum gain is exploited to mitigate the interference among MSNs and also from external sources. Meanwhile, since the AOAs are estimated based on the array signals collected by multiple antennas, the received SNR is increased significantly by the array gain. Therefore, ATML can achieve high localization accuracy with low transmission power to save the energy consumption on MSNs.
- MSNs broadcast pre-assigned spreading sequences simultaneously and ANs separate the signals based on the signal orthogonality. The relative positions of multiple MSNs can be determined at the same time. Hence, ATML has a high speed and efficiency for multi-target localization, which is critical for the industrial applications with stringent latency requirements.

The remainder of this paper is organized as follows. In Section 2, we briefly review the node localization and AOA estimation technologies. Section 3 introduces the system model of the designed SIWSN architecture. The ATML scheme is proposed in Section 4 including the communication system architectures on both MSNs and ANs, signal separation based on CDMA, propagation path parameter estimation, and multi-target localization. Section 5 presents the simulation results to evaluate and compare the performance of ATML. The testbed implementation and experiments are presented in Section 6. Section 7 concludes the paper and points out the future research issues.

## 2. Related Work

Mao et al. [7] has made a comprehensive summary of node positioning algorithms that can be distinguished into two main categories: range-based scheme and range-free scheme. Extensive analysis and comparison have been reported in the literature.

The range-based schemes usually use the trilateration and triangulation positioning methods by accurately measuring the distance or angle information between nodes. These kinds of schemes commonly measure the received signal strength indicator (RSSI) [8,9], AOA [10,11,12], time of arrival (TOA) [13], and time difference of arrival (TDOA) [14]. Although the RSSI methods are most easily realized by using only the signal strength information, they have greater instability and larger errors in complex propagation environments. Chen et al. [13] used two generalized geometrical positioning algorithms based on TOA measurements without time synchronization. The TDOA technology usually uses ultrasound for measurement. Shi et al. [14] formulated the localization problem as a nonconvex optimization problem and adopted semi-definite relaxation to obtain a convex optimization problem. The scheme could minimize the worst-case position estimation error.

The AOA-based localization does not need synchronization among nodes and also has relatively high precision. Amundson et al. [10] proposed a localization and navigation system using radio interferometric AOA estimation implemented for wireless sensor nodes. However, the scheme is limited to a single dimension and susceptible to multipath effect. Wielandt et al. [11] presented an angle of arrivation (ACF) positioning scheme that utilized the tag signal AOA and the distance estimated by the power control method to locate active tags. Wielandt et al. [12] proposed a new AOA-based approach that applied the spatial smoothing in pre-processing and computed the reference vectors. This approach was experimentally evaluated in LOS and non-LOS (NLOS) conditions. The hybrid approaches combining various range estimations together to improve localization accuracy have also been studied. Tomic et al. [8] proposed to track a signal-emitting mobile target with RSSI and AOA measurements. In [9], Tomic et al. proposed another scheme based on RSSI and AOA measurements for localization in WSNs. Obviously, AOA estimation is critical in these range-based methods. The traditional AOA estimation methods include MUSIC [15], ESPRIT [16], CLEAN [17], SAGE [18], and SPASE [19]. Alsadoon et al. [20] presented a new method with significantly reduced complexity by avoiding constructing the covariance matrix and computing the matrix inverse or applying eigenvalue decomposition.

The range-based schemes are usually affected significantly by the multipath propagation effect and the NLOS problem, which cause significant measurement errors. Ziolkowski et al. [21] analyzed the influence of environment transmission properties on the signal reception angular spread based on measurement results. It was shown that spatial averaging could provide a better estimation of the reception angle. They also studied the limitations in the direction-finding process of radio sources with directional antennas in an urbanized environment [22]. The results could help to correct the bearing error to improve the localization efficiency. For the NLOS scenario, Shi et al. [23] proposed a maximum likelihood estimator (MLE) that utilized all the available measurements and explicitly took the probabilities of occurrences of LOS and NLOS propagations into account.

The range-free methods are based on the number of hops between two communicating nodes as the distance metric. The classical range-free methods include the centroid-based, amorphous, and distance vector hop (DV-Hop) based algorithms [24,25]. Among these, the DV-Hop scheme is most widely used due to high positioning accuracy in isotropic networks. However, it has a lower accuracy in asymmetric and sparse networks. Without extra hardware and measurement, the range-free methods are not very accurate compared with the range-based schemes.

## 3. Network Model

**Figure 1:**
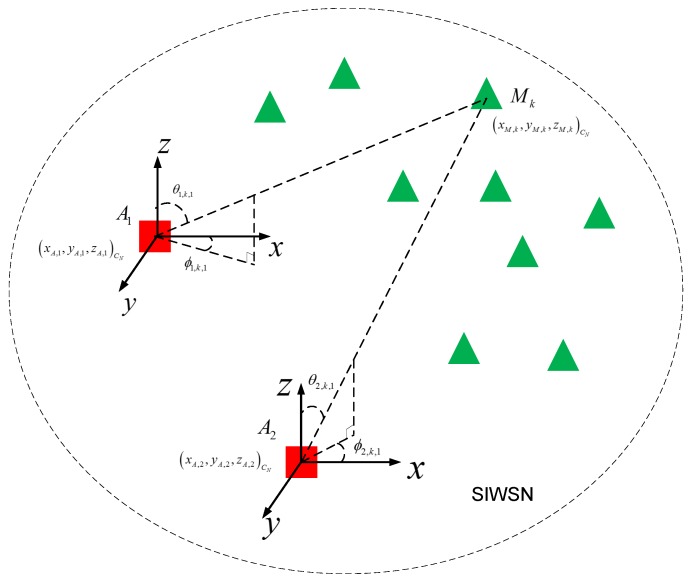
The network model of the ATML scheme for node localization in a SIWSN.

In the ATML scheme, we employ two ANs to determine the 3D relative positions of a number of MSNs in a SIWSN. Let $A_{1}$ and $A_{2}$ denote the two ANs. Suppose that there are *K* MSNs in the network, denoted by $M_{k}\left(k=1,2,\cdots ,K\right)$. The positions of the ANs are fixed and pre-determined. The network model is illustrated in Figure 1.

**Figure 2:**
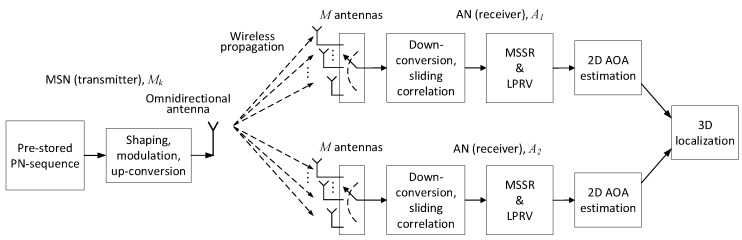
The MT-SIMO signal flow on the MSNs and ANs in the ATML scheme for 3D node localization.

The architectures and signal flow of the MT-SIMO scheme on the MSNs and ANs are shown in Figure 2. Each MSN uses an omnidirectional antenna to broadcast its signals, while an AN receives the signals from all the MSNs with an antenna array. Suppose that each AN array comprises *M* antenna elements (for easy presentation, we assume that the antenna arrays on both ANs have the same structure, but this is unnecessary and different 2D or crown arrays may be employed). In order to perform 3D localization, the AN antennas should be sector/directional antennas and placed in 2D structures (e.g., rectangular or “L”-shaped planar arrays) or 3D structures (e.g., cylindrical or crown arrays). The planar arrays can cover a semisphere because the main lobes of the antennas are all pointing to one side of the array. The cylindrical or crown arrays can cover a whole sphere because the main lobes point to all the directions. The structure of the antenna arrays can be selected according to the deployment of the network. If all the MSNs are located on one side of an AN, we can adopt the planar arrays. However, if the MSNs are located in all directions to an AN, the cylindrical or crown arrays should be adopted. Thus, all the MSNs are visible to the AN antennas and in main lobes.

There is a common orthogonal coordinate system for the network, which is denoted by $C_{N}$. The position of $M_{k}$ in $C_{N}$ is ${\left(x_{M,k},y_{M,k},z_{M,k}\right)}_{C_{N}}$. The positions of the two ANs are ${\left(x_{A,i},y_{A,i},z_{A,i}\right)}_{C_{N}}$ for $A_{i}$ (i=1,2). Furthermore, we adopt the spherical coordinate systems for the MSN positions with respect to $A_{1}$ and $A_{2}$, which are denoted by $C_{1}$ and $C_{2}$, respectively. The coordinate systems are defined based on the body frames of the two ANs. The origins are located at the centers of the antenna arrays of the ANs. The *x*–*y* and *x*–*z* planes correspond to the azimuth and elevation dimensions, respectively, as shown in Figure 1.

In complicated scattering industrial environments, signals generally propagate from an MSN to an AN through multiple paths, resulting in the *multipath components (MPCs)*. Suppose that there are $L_{i,k}$ propagation paths from $M_{k}$ to $A_{i}$. The parameter set of the *l*-th ($l=1,2,\cdots ,L_{i,k}$) component received on the antennas on $A_{i}$ is [26]

(1) $$\omega_{i,k,l}=\left\{\alpha_{i,k,l},\tau_{i,k,l},\phi_{i,k,l},\theta_{i,k,l},\nu_{i,k,l}\right\},$$

where $\alpha_{i,k,l}$, $\tau_{i,k,l}$, $\phi_{i,k,l}$, $\theta_{i,k,l}$, and $\nu_{i,k,l}$ are the complex amplitude (including the path loss and phase shift), TOA (i.e., propagation delay), azimuth of arrival, elevation of arrival, and Doppler frequency, respectively.

It is important to note that the ATML scheme requires propagation along the connection directions from each MSN to both ANs. This condition can be satisfied in real industrial environments if there are clear LOS paths or LOS paths blocked by non-metallic materials such as concrete walls and plastic or wood cargoes. The radio signals can penetrate the obstacles and propagate along the LOS directions. For example, the radio propagation penetrating a building is illustrated in [26]. If the LOS paths are blocked by metal materials, the radio signals cannot penetrate the objects. Then, we can deploy more ANs in the environment such that all the MSNs have LOS propagation to two ANs. Deploying more ANs results in a higher cost, but the NLOS problem can be avoided.

The propagation delay along the LOS path is generally smaller than the MPCs between a pair of Tx and Rx. Hence, the first arriving component (l=1) travels through the LOS path with the parameter set of $\omega_{i,k,1}$. The azimuth and elevation direction from $A_{i}$ to $M_{k}$ is $\phi_{i,k,1}$ and $\theta_{i,k,1}$, respectively, as shown in Figure 1. Thus, the relative position of $M_{k}$ with respect to $A_{i}$ in the coordinate system of $C_{i}$ is $\left(d_{i,k},\phi_{i,k,1},\theta_{i,k,1}\right)$ where $d_{i,k}$ is the distance. Our purpose is to determine the 3D positions of the *K* MSNs in the common network coordinate system, ${\left(x_{M,k},y_{M,k},z_{M,k}\right)}_{C_{N}}$ for k=1,2,⋯,K, simultaneously and accurately. In practical multipath scattering channels, several sub-paths may arrive at the Rx at very close delays (in a small delay bin). These paths belong to a cluster in the delay domain. Because the ATML scheme utilizes the incident angles of the LOS paths for node localization and the parameters of the MPCs are not used, the clustering behavior of the MPCs can be ignored.

## 4. Multi-Target Detection and Localization

In this section, the ATML scheme that detects the signals from MSNs and locates their positions in a SIWSN is presented. The MT-SIMO signal transmission/detection system is first designed; then, the propagation parameter (especially the 2D AOA) estimation based on the spreading-sequence response using the MLE is proposed, and finally the relative 3D position of an MSN is deduced.

### 4.1. MT-SIMO Signal Transmission

We utilize the CDMA technology to realize the multi-target detection (separating the signals from different MSNs). Each MSN is assigned a pre-defined spread spectrum sequence, i.e., a PN-sequence with the length of *N* chips. The spreading sequence assigned to $M_{k}$ is denoted by $u_{k}\left(\tau \right)$ and expressed as

(2) $$u_{k}\left(\tau \right)=\sum_{n=1}^{N}c_{k,n}\mathrm{rect}\left(\tau -n\Delta_{c}\right),\;c_{k,n}\in \left\{+1,-1\right\},$$

where $\mathrm{rect}\left(\tau \right)$ denotes the rectangular pulse of a chip that has the duration of $\Delta_{c}$ and power of $P_{c}=\int_{0}^{\Delta_{c}}{\mathrm{rect}}^{2}\left(\tau \right)d\tau$. The duration of the spreading sequence is $T_{u}=N\Delta_{c}$. The sequences of the *K* MSNs are orthogonal and the auto-correlation functions are pulse functions. Hence, the cross-correlation functions of the spreading sequences are [27]

(3) $$\langle u_{k_{1}}\left(\tau \right),\;u_{k_{2}}\left(\tau \right)\rangle =u_{k_{1}}\left(\tau \right)*u_{k_{2}}^{*}\left(-\tau \right)=\left\{\begin{array}{cc} \varepsilon_{k_{1},k_{2}}, & k_{1}\neq k_{2},\\ NP_{c}\delta \left(\tau \right), & k_{1}=k_{2}, \end{array}\right.$$

where <·,·> denotes the sliding inner-production, $\varepsilon_{k_{1},k_{2}}$ is a very small number $\varepsilon_{k_{1},k_{2}}\to 0$ and δ(τ) is the delta function. The peak amplitude of the delta function is $\int_{0}^{T_{u}}u_{k}^{2}\left(\tau \right)d\tau =NP_{c}$.

An MSN modulates (e.g., via binary phase shift keying, BPSK) the carrier with its spreading sequence circularly and continuously and uses an omnidirectional antenna to transmit the modulated signal. The MSNs can transmit asynchronously, and each AN captures the signals using an *M* element antenna array, as mentioned in Section 3. An AN keeps receiving signals for the duration of more than $2T_{u}$ such that it can capture at least one complete spreading sequence from each MSN. To reduce the hardware complexity and cost of the ANs, the signal reception can be realized in a time-division-multiplexing (TDM) manner. An AN uses only a single radio and baseband processing chain. The antennas of the array are connected to an *M*-input-1-output microwave switch. The switch connects one receiving antenna for more than $2T_{u}$ to capture the signals in the air, and then changes to the next antenna. Consequently, the antennas receive the signals one-by-one controlled by the microwave switch. After the time of $2MT_{u}$, all the antennas receive at least one complete spreading sequence from every MSN. As long as the antenna switching is fast enough, the movement of the MSNs during $2MT_{u}$ is ignorable and the signals on the receiving antennas can be regarded as being captured simultaneously. The MT-SIMO links between an MSN and the ANs and the signal flow are illustrated in Figure 2. Please note that synchronization and coordination among the MSNs are not required.

As mentioned in Section 3, there are $L_{i,k}$ propagation paths from $M_{k}$ to $A_{i}$. Hence, $L_{i,k}$ MPCs are received on each antenna. In this system, the pattern of one antenna element in an array refers to the complex radiation gain with respect to the reference element (e.g., the first antenna) for a given azimuth and elevation direction. The pattern of the *m*-th (m=1,2,⋯,M) antenna of $A_{i}$ is denoted by $s_{i,m}\left(\phi ,\theta \right)$ where (ϕ,θ) is the 2D radiation/incident direction. The amplitude of $s_{i,m}\left(\phi ,\theta \right)$ is the scale of the signal magnitude by the antenna gain. The phase of $s_{i,m}\left(\phi ,\theta \right)$ is the phase difference between the signals impinging at the *m*-th antenna and the reference antenna. When the *l*-th path from $M_{k}$ arrives at $A_{i}$ at the 2D incident angle of $\left(\phi_{i,k,l},\theta_{i,k,l}\right)$, the pattern of the *m*-th antenna will be $s_{i,m}\left(\phi_{i,k,l},\theta_{i,k,l}\right)$. Thus, the baseband signal from $M_{k}$ received on the *m*-th antenna on $A_{i}$ through the *l*-th path at the moment of *t* is denoted by $y_{i,m,k,l}\left(\tau \right)$. The variable τ is the excess delay of the propagation paths. The receiving time, *t*, is a parameter of the signal and ignored in the notations for easy presentation. Thus, the received signal is

(4) $$y_{i,m,k,l}\left(\tau \right)=\alpha_{i,k,l}s_{i,m}\left(\phi_{i,k,l},\theta_{i,k,l}\right)e^{j2\pi \nu_{i,k,l}t}u_{k}\left(\tau -\tau_{i,k,l}\right),$$

where $u_{k}\left(\tau -\tau_{i,k,l}\right)$ is the circular shift of $u_{k}\left(\tau \right)$ by the propagation delay. As all of the *K* MSNs are broadcasting simultaneously, the signals received on the AN antennas are the superposition of all the MSNs’ signals. The received (observed) signal on the *m*-th antenna on $A_{i}$ is

(5) $${\hat{y}}_{i,m}\left(\tau \right)=\sum_{k=1}^{K}\sum_{l=1}^{L_{i,k}}y_{i,m,k,l}\left(\tau \right)+w_{i,m}\left(\tau \right),$$

where $w_{i,m}\left(\tau \right)$ is the noise in the *m*-th receiving chain on $A_{i}$.

By organizing the radiation gains of all the *M* antennas pointing to the 2D AOA (ϕ,θ) in one vector, we can obtain the *steering vector* of the antenna array on $A_{i}$ pointing to the direction, which is

(6) $$s_{i}\left(\phi ,\theta \right)={\left[\begin{array}{ccccc} s_{i,1}\left(\phi ,\theta \right) & s_{i,2}\left(\phi ,\theta \right) & s_{i,3}\left(\phi ,\theta \right) & \cdots & s_{i,M}\left(\phi ,\theta \right) \end{array}\right]}^{T}.$$

It is important to note that the antenna pattern, $s_{i,m}\left(\phi ,\theta \right)$, is a parameter matrix that is measured in an anechoic chamber. The antenna gains for all the 2D directions of $\phi \in [0^{\circ },360^{\circ })$ and $\theta \in [0^{\circ },180^{\circ }]$ are all measured. For example, when the antenna pattern is measured with the step of $1^{\circ }$, the matrix dimension is 360×181 and the elements in the matrix are the complex radiation gain with respect to the reference antenna.

### 4.2. Multi-Target Detection

We can separate and detect each MSN’s signal based on the orthogonality among the spreading sequences. The ANs perform sliding-correlations between the received signal ${\hat{y}}_{i,m}\left(\tau \right)$ and the spreading sequence $u_{k}\left(\tau \right)$ in the delay domain to extract the signal from $M_{k}$. The observed result is given as

(7) $$\begin{array}{cc} {\hat{h}}_{i,m,k}\left(\tau \right) & =\langle {\hat{y}}_{i,m}\left(\tau \right),\;u_{k}\left(\tau \right)\rangle =\left[\sum_{k=1}^{K}\sum_{l=1}^{L_{i,k}}y_{i,m,k,l}\left(\tau \right)+w_{i,m}\left(\tau \right)\right]*u_{k}^{*}\left(-\tau \right)\\ & =\sum_{l=1}^{L_{i,k}}y_{i,m,k,l}*u_{k}^{*}\left(-\tau \right)+\sum_{k^{\prime }=1,k^{\prime }\neq k}^{K}\sum_{l=1}^{L_{i,k^{\prime }}}y_{i,m,k^{\prime },l}*u_{k}^{*}\left(-\tau \right)+w_{i,m}\left(\tau \right)*u_{k}^{*}\left(-\tau \right). \end{array}$$

By substituting Equations (3) and (4) into Equation (7), we can obtain

(8) $$\begin{array}{cc} {\hat{h}}_{i,m,k}\left(\tau \right) & =\sum_{l=1}^{L_{i,k}}NP_{c}\alpha_{i,k,l}s_{i,m}\left(\phi_{i,k,l},\theta_{i,k,l}\right)e^{j2\pi \nu_{i,k,l}t}\delta \left(\tau -\tau_{i,k,l}\right)\\ & \;+\sum_{k^{\prime }=1,k^{\prime }\neq k}^{K}\sum_{l=1}^{L_{i,k^{\prime }}}\alpha_{i,k^{\prime },l}s_{i,m}\left(\phi_{i,k^{\prime },l},\theta_{i,k^{\prime },l}\right)e^{j2\pi \nu_{i,k^{\prime },l}t}\varepsilon_{k,k^{\prime }}+w_{i,m}^{\prime }\left(\tau \right)\\ & =\sum_{l=1}^{L_{i,k}}A_{i,m,k,l}\delta \left(\tau -\tau_{i,k,l}\right)+\zeta_{i,m,k}\left(\tau \right)+w_{i,m}^{\prime }\left(\tau \right), \end{array}$$

where $A_{i,m,k,l}=NP_{c}\alpha_{i,k,l}s_{i,m}\left(\phi_{i,k,l},\theta_{i,k,l}\right)e^{j2\pi \nu_{i,k,l}t}$ is the complex amplitude of the *l*-th peak in the cross-correlation function and

(9) $$\zeta_{i,m,k}\left(\tau \right)=\sum_{k^{\prime }=1,k^{\prime }\neq k}^{K}\sum_{l=1}^{L_{i,k^{\prime }}}\alpha_{i,k^{\prime },l}s_{i,m}\left(\phi_{i,k^{\prime },l},\theta_{i,k^{\prime },l}\right)e^{j2\pi \nu_{i,k^{\prime },l}t}\varepsilon_{k,k^{\prime }}\approx 0$$

is the inter-MSN interference received on the *m*-th antenna on $A_{i}$. Because of the correlation property of the spreading sequences, the cross-correlation result in Equation (8) generates $L_{i,k}$ peaks at the excess delays of $\tau_{i,k,l}$ with the observed complex amplitude of

(10) $${\hat{A}}_{i,m,k,l}=A_{i,m,k,l}+\zeta_{i,m,k}\left(\tau_{i,k,l}\right)+w_{i,m}^{\prime }\left(\tau_{i,k,l}\right),$$

for $l=1,2,\cdots ,L_{i,k}$. In this work, ${\hat{h}}_{i,m,k}\left(\tau \right)$ is called the multipath spreading sequence response (MSSR).

For the signals received on the AN antennas, ${\hat{y}}_{i,m}\left(\tau \right)$, we can perform sliding-correlation using the *K* spreading sequences, $u_{k}\left(\tau \right)$ for k=1,2,⋯,K. Thus, the MSSR for each MSN, ${\hat{h}}_{i,m,k}$, is observed on every receiving antenna. The interference from other MSNs and also external sources is removed by the sliding correlation. Hence, the MT-SIMO scheme provides the advantages of mitigating interference and high signal-to-interference-ratio (SIR), resulting in the spread spectrum gain.

### 4.3. Iterative ML Estimation of AOA

The localization of an MSN is performed based on the LOS path AOAs from the MSN to both ANs. As discussed in the previous subsection, based on the observed MSSRs of $M_{k}$ on the AN arrays, we can distinguish the LOS paths from the other reflected/scattered MPCs in a complicated industrial environment. In this subsection, we propose a simple ML method to estimate the AOAs of the LOS paths.

According to Equation (8), the peak of the *l*-th path occurs at the excess delay of $\tau_{i,k,l}$ in all the MSSRs on the antennas on $A_{i}$. The observed amplitudes of the LOS path (the first peak) on all the antennas (m=1,2,⋯,M) are organized in the vector of

(11) $${\hat{a}}_{i,k}={\left[\begin{array}{ccccc} {\hat{A}}_{i,1,k,1} & {\hat{A}}_{i,2,k,1} & {\hat{A}}_{i,3,k,1} & \cdots & {\hat{A}}_{i,M,k,1} \end{array}\right]}^{T}.$$

The observation vector, ${\hat{a}}_{i,k}$, is called the *LOS path response vector (LPRV)*. It is worth noticing that, as given in Equations (8) and (10), the observed peak amplitudes in ${\hat{a}}_{i,k}$ contain the noise, interference, and also the AOA information of the LOS path, $\left(\phi_{i,k,1},\theta_{i,k,1}\right)$. Our object is to estimate the AOA for localization from ${\hat{a}}_{i,k}$. The iterative MLE is given as follows.

1. Set the initial iteration index, g=1.
2. Set the initial elevation AOA of the LOS path from $M_{k}$ to $A_{i}$ as ${\tilde{\theta }}_{i,k,1}^{\left(g\right)}=90^{\circ }$.
3. The incident azimuth angle is estimated based on the antenna array steering vectors. ${\tilde{\phi }}_{i,k,1}^{\left(g\right)}$ is updated by
  (12) $${\tilde{\phi }}_{i,k,1}^{\left(g\right)}=\underset{\phi_{i,k,1}\in \left[0,360^{\circ }\right)}{\arg\max}\left|s_{i}{\left(\phi_{i,k,1},{\tilde{\theta }}_{i,k,1}^{\left(g\right)}\right)}^{H}{\hat{a}}_{i,k}\right|.$$
4. By using ${\tilde{\phi }}_{i,k,1}^{\left(g\right)}$, the incident elevation angle, ${\tilde{\theta }}_{i,k,1}^{\left(g\right)}$, is updated by
  (13) $${\tilde{\theta }}_{i,k,1}^{\left(g+1\right)}=\underset{\theta_{i,k,1}\in \left[0,180\right]}{\arg\max}\left|s_{i}{\left({\tilde{\phi }}_{i,k,1}^{\left(g\right)},\theta_{i,k,1}\right)}^{H}{\hat{a}}_{i,k}\right|.$$
5. Update the iteration index by g=g+1.
6. Repeat Steps 3 to 5, until the results converge by satisfying the condition of
  (14) $$\sqrt{{\left({\tilde{\phi }}_{i,k,1}^{\left(g-1\right)}-{\tilde{\phi }}_{i,k,1}^{\left(g-2\right)}\right)}^{2}+{\left({\tilde{\theta }}_{i,k,1}^{\left(g\right)}-{\tilde{\theta }}_{i,k,1}^{\left(g-1\right)}\right)}^{2}}\leq \xi ,$$
  where ξ is a small number.

For the first time using Equation (12), we have set ${\tilde{\theta }}_{i,k,1}^{\left(1\right)}=90^{\circ }$ in Step 2. ${\hat{a}}_{i,k,1}$ is the observation of the antenna array. $s_{i}\left(\phi_{i,k,1},{\tilde{\theta }}_{i,k,1}^{\left(1\right)}\right)$ is the antenna array pattern and has been measured in a chamber. We can obtain the value of $s_{i}\left(\phi_{i,k,1},{\tilde{\theta }}_{i,k,1}^{\left(1\right)}\right)$ with $\phi_{i,k,l}\in \left[0^{\circ },360^{\circ }\right)$ and ${\tilde{\theta }}_{i,k,1}^{\left(1\right)}=90^{\circ }$. Thus, we can calculate the right side of Equation (12) for $\phi_{i,k,l}\in \left[0^{\circ },360^{\circ }\right)$. Finally, we can find out the maximum value of the right side, and the corresponding $\phi_{i,k,l}$ is the updated value. Similarly, for Equation (13), we can obtain the value of $s_{i}\left({\tilde{\phi }}_{i,k,1}^{\left(g\right)},\theta_{i,k,1}\right)$ where ${\tilde{\phi }}_{i,k,1}^{\left(g\right)}$ has been determined in the previous step and $\theta_{i,k,1}\in \left[0^{\circ },180^{\circ }\right]$. Thus, we can find out the maximum value of the right side for $\theta_{i,k,1}\in \left[0^{\circ },180^{\circ }\right]$ and the corresponding $\theta_{i,k,l}$.

### 4.4. Localization of MSNs

As shown in the network model of Figure 1, the target MSN, $M_{k}$, should be on the line through $A_{i}$ with the direction of $\left({\tilde{\phi }}_{i,k,1},{\tilde{\theta }}_{i,k,1}\right)$ in the spherical coordinate system $C_{i}$ where i=1,2 for the two ANs. The two lines are denoted by $f_{i,k}{\left(x,y,z\right)}_{C_{N}}$ in the common network coordinate system, $C_{N}$. Ideally, $M_{k}$ is located at the intersection of $f_{1,k}$ and $f_{2,k}$. However, in practical environments, the two lines may have no point of intersection (not in the same plane) due to measurement errors caused by noise and interference, and they are *skew lines*. How to determine the position of $M_{k}$ according to 3D Euclidean geometry is described below.

At first, we convert the definitions of the two lines from the spherical coordinate systems (for AOA estimation) to the network orthogonal coordinate system (for target localization). The intersection between the line $f_{i,k}{\left(x,y,z\right)}_{C_{N}}$ and the unit sphere is denoted by $P_{i,k}$. In $C_{i}$, the coordinate of $P_{i,k}$ is

(15) $$P_{C_{i},i,k}=\left[\begin{array}{c} x_{C_{i},P_{i,k}}\\ y_{C_{i},P_{i,k}}\\ z_{C_{i},P_{i,k}} \end{array}\right]=\left[\begin{array}{c} \sin\theta_{i,k,1}\cos\phi_{i,k,1}\\ \sin\theta_{i,k,1}\sin\phi_{i,k,1}\\ \cos\theta_{i,k,1} \end{array}\right].$$

Thus, the coordinates of $P_{i,k}$ in $C_{N}$ are $P_{C_{N},i,k}=P_{C_{i},i,k}+A_{C_{N},i},$ where $A_{C_{N},i}$ are the coordinates of $A_{i}$ in $C_{N}$. Consequently, the two lines are defined by the two pairs of points, $A_{C_{N},i}$ and $P_{C_{N},i,k}$, in $C_{N}$ for i=1,2.

The two pairs of points define a tetrahedron in 3D space as the four vertices. The volume of the tetrahedron is

(16) $$V=\frac{1}{6}\left|\det\left[\begin{array}{c} P_{C_{N},1,k}-A_{C_{N},1}\\ A_{C_{N},1}-A_{C_{N},2}\\ A_{C_{N},2}-P_{C_{N},2,k} \end{array}\right]\right|,$$

where det[·] is the determinant. The relationship between the two lines is discussed in the following two cases.

- If the volume of the tetrahedron is zero (i.e., V=0), the two lines are coplanar and have an intersection. The intersection point can be easily determined by $f_{i,k}{\left(x,y,z\right)}_{C_{N}}$ in the plane.
- If the volume of the tetrahedron is nonzero (i.e., V>0), $f_{1,k}$ and $f_{2,k}$ are skew lines without intersection. The points on the line segment perpendicular to both lines have the minimum sum of distances to $f_{1,k}$ and $f_{2,k}$. Hence, the MSN position is approximated by the midpoint of the line segment.
  First, we define the direction vectors of the two lines by $D_{C_{N},i,k}=P_{C_{N},i,k}-A_{C_{N},i}$ for i=1,2. Thus, the two lines can be expressed as $Q_{C_{N},i,k}=A_{C_{N},i}+\lambda_{i}D_{C_{N},i,k}$ where $Q_{C_{N},i,k}$ represents an arbitrary point on the line with the real number $\lambda_{i}$ determining where the point is on the line. Then, the point on $f_{1,k}$ nearest to $f_{2,k}$ is given by
  (17) $$Q_{C_{N},1,k}^{*}=P_{C_{N},1,k}+\frac{\left(P_{C_{N},2,k}-P_{C_{N},1,k}\right)\cdot V_{C_{N},2}}{D_{C_{N},1}\cdot V_{C_{N},2}}\times D_{C_{N},1}.$$
  In Equation (17), $V_{C_{N},2}=D_{C_{N},2}\times \left(D_{C_{N},1}\times D_{C_{N},2}\right)$ where × is cross product. Similarly, the point on $f_{2,k}$ nearest to $f_{1,k}$ is given by
  (18) $$Q_{C_{N},2,k}^{*}=P_{C_{N},2,k}+\frac{\left(P_{C_{N},1,k}-P_{C_{N},2,k}\right)\cdot V_{C_{N},1}}{D_{C_{N},2}\cdot V_{C_{N},1}}\times D_{C_{N},2},$$
  where $V_{C_{N},1}=D_{C_{N},1}\times \left(D_{C_{N},2}\times D_{C_{N},1}\right)$. Thus, $Q_{C_{N},1,k}^{*}$ and $Q_{C_{N},2,k}^{*}$ form the shortest line segment joining $f_{1,k}$ and $f_{2,k}$. Finally, the location of $M_{k}$ can be determined as
  (19) $$P_{C_{N},M_{k}}=\frac{Q_{C_{N},1,k}^{*}+Q_{C_{N},2,k}^{*}}{2}.$$

Since we have determined the coordinates of the target MSN, the distance $d_{i,k}$ is readily calculated from its coordinate.

## 5. Simulation Results

### 5.1. Simulation Setting

We have conducted extensive simulations to evaluate and compare the performance of ATML with other methods. The simulations are performed using MATLAB (R2016b). The ANs are equipped with square arrays with the dimension of M×M. The ideal radiation pattern (steering vectors) of the antenna arrays are adopted. The phase differences among the antenna elements are theoretical and the errors/deviations of the antenna gains are not considered. Suppose that the spacing between adjacent rows and columns is *d*. We use the antenna at the position of (1,1) as the reference point. Then, for the incident direction of (ϕ,θ), the radiation pattern of the antenna at the position of (m,n) in the array is $s_{i,(m,n)}=md\cos\theta \cos\phi +nd\cos\theta \sin\phi$.

The system parameters are listed in Table 1. Because the chip rate is 20 Mcps (chips per second), the chip duration equals 50 ns (the reciprocal of the chip rate) and the bandwidth is 20 MHz. The PN-sequences are transmitted circularly and received by the antenna arrays on the two ANs. The signals are superimposed with additive white Gaussian noise (AWGN) for various SNRs.

### 5.2. Multipath Signal Detection and AOA Estimation

We focus on the signal transmission and evaluate the AOA estimation between a pair of MSN and AN in this subsection. In the simulation, the number of propagation paths between the MSN and AN is assumed to be L=5. The path parameters are selected randomly and uniformly in the corresponding ranges and listed in Table 2. We clarify that the unit of the propagation delay is chips (with the time duration of one chip in the PN-sequence). The parameter $|\alpha_{l}{|}^{2}$ is the received path power. In the simulations, the phase shifts caused by the propagation through paths are ignored (set as zero) because the AOA estimation is performed based on the phase differences of one path on the antenna elements. The received signal is constructed by the multipath superposition according to the signal model given in Equations (4) and (5).

**Figure 3:**
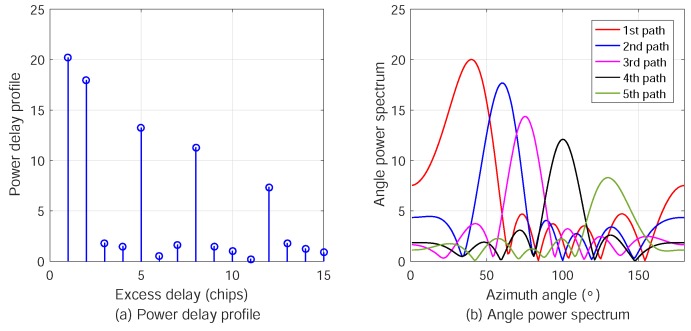
Power delay profile and angular power spectra of the five MPCs between the MSN and AN.

**Figure 4:**
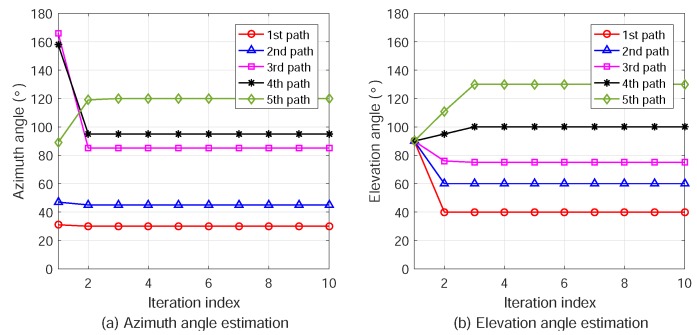
Iterative ML estimation of azimuth and elevation AOAs of multiple propagation paths.

The power delay profile (PDP) and angular power spectra (APS) of the five MPCs are shown in Figure 3. We can see that the five significant MPCs and the small pulses at other excess delays are caused by the AWGN in constructing the received signal. To estimate the AOA of the LOS path, the updated values of the azimuth and elevation angles in the iterations are plotted in Figure 4. It is shown that $\phi_{1}$ and $\theta_{1}$ converge the correct values after three iterations. The spread spectrum gain and antenna array gain provide the relatively high signal to interference plus noise ratio (SINR), resulting in fast convergence and high accuracy. Considering the complexity of the multipath spatial propagation in radio channels, the spread spectrum scheme in ATML can effectively mitigate the multipath effect by extracting the LOS component for localization.

### 5.3. Multi-Target AOA Estimation

**Figure 5:**
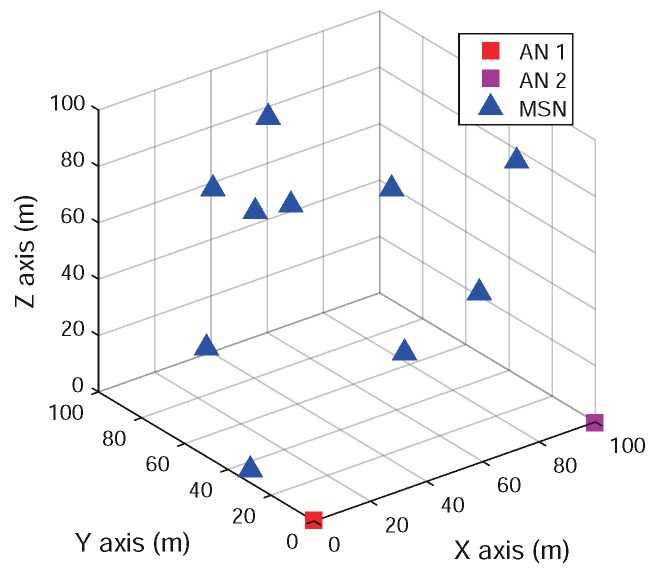
The network coordinate system and topology when there are K=10 MSNs.

The AOA estimation for multiple targets is simulated for relative position determination. We consider an SIWSN comprised of two pre-positioned ANs and a varying number of MSNs. The MT-SIMO signals as described above are used. The positions of MSNs are randomly distributed in a cube of 100×100×100 m^3^. For example, when the number of MSNs is K=10, the network coordinate system and the topology are shown in Figure 5. The ANs are placed at the coordinates of (0,0,0) and (100,0,0) m. The two ANs should be placed by the two sides of the deployment area of MSNs to obtain the best performance. The simulation results for this typical scenario are presented in this subsection.

**Figure 6:**
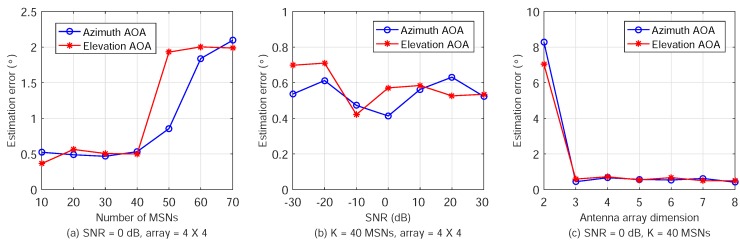
AOA estimation results for various number of MSNs, SNR, and antenna array dimension.

The ML estimation errors of the azimuth and elevation angles are plotted in Figure 6. First, Figure 6a shows the results when the number of MSNs, *K*, increases from 10 to 70. The received SNR is set as 0 dB and 4×4 antenna arrays are employed on the ANs. The AOA estimation errors increase slightly because the mutual interference among the MSNs is more severe. However, we can see that the maximum error is about $2^{\circ }$ even when there are K=70 MSNs. This indicates that the ATML scheme is effective in positioning a large number of MSNs simultaneously in WSNs.

Second, the estimation errors with varying SNR are plotted in Figure 6b, where the received SNR increases from −30 to 30 dB with steps of 10 dB each. With each SNR, K=40 MSNs are placed randomly and the antenna array dimensions are still 4×4. It is shown that the AOA estimation errors are mostly around $0.5^{\circ }$. This indicates that the ATML scheme has good robustness and high accuracy for varying SNR. This is mainly due to, as mentioned earlier, exploiting the spread spectrum gain and antenna array gain.

Third, the estimation errors using different sizes of antenna arrays on ANs are plotted in Figure 6c. The array dimension is changed from 2×2 (4 antenna elements) to 8×8 (64 antenna elements). The received SNR is set as 0 dB and number of MSNs is K=40. We can see that the estimation errors are about $8^{\circ }$ when the array dimension is 2×2. This is because the aperture antenna arrays is too small and there are not sufficient antenna elements in the azimuth and elevation dimensions. The ML method cannot provide accurate AOA estimation with insufficient information. However, the errors reduce quickly to only $0.5^{\circ }$ when the array dimension is increased to 3×3. Therefore, ATML can work even using small antenna arrays on ANs, requiring low hardware complexity and cost.

### 5.4. Multi-Target Localization

**Figure 7:**
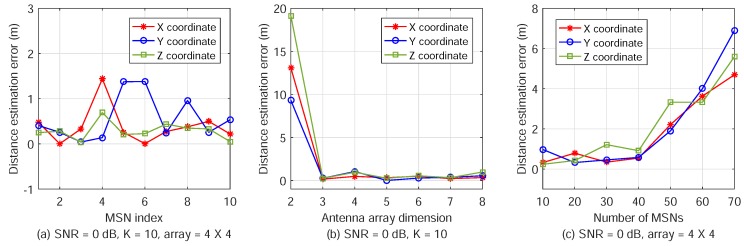
Multi-target position estimation for various numbers of MSNs, SNR, and antenna array dimension.

In this subsection, we randomly deploy 10 MSNs and estimate their positions using ATML. The estimation errors in the *x*, *y*, and *z* coordinates are plotted in Figure 7.

Figure 7a shows the coordinate estimation errors of the 10 MSNs when the antenna array dimension is 4×4 and the received SNR is 0 dB. The *x*-label is the index of the MSNs, from the 1st to the 10th MSN. It can be seen that the errors are mostly about 0.5 m and the maximum value is 1.5 m.

In Figure 7b, the received SNR is set as 0 dB and the number of MSNs is K=40. The antenna array dimension is set from 2×2 to 8×8. The plotted results are the average of the position estimation errors of all MSNs. To obtain the average result, we sum up the *x*-coordinate errors of all the *K* MSNs together and then divide the summation by *K*. The average results of the *y* and *z*-coordinates are obtained in the same way. When the array dimension is 2×2, the large position estimation errors are caused by the large AOA estimation errors due to insufficient antenna numbers, as shown in Figure 6c. When the array dimension is greater than 3×3, we can obtain accurate estimation results.

In Figure 7c, the number of MSNs varies from K=10 to 70 with a step size of 10. The received SNR is set as 0 dB and the array dimension is 4×4. The results are the average of the position estimation errors of the 40 MSNs. The estimation errors increase with the number of MSNs when *K* is larger than 40 due to severe inter-MSN interference. For a large number of MSNs to locate, we can use larger antenna arrays on ANs (to increase *M*) or longer PN-sequences with more chips (to increase *N*). When the SINR is increased, more MSNs can be located simultaneously and accurately.

**Figure 8:**
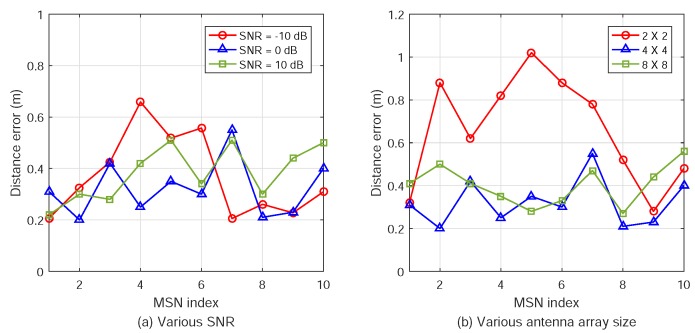
Multi-target position estimation RMSs for various SNR and antenna array dimension.

Furthermore, the square root errors (RMSs) of the MSN positions are plotted in Figure 8.

We performed simulations to compare the proposed ATML scheme with other algorithms in the literature. In [28], the localization scheme, AOA Localization with RSSI Differences of Directional Antennas (ALRD), was presented. ALRD fits RSSI values into a parabola function of AOA between $0^{\circ }$ and $90^{\circ }$ by quadratic regression analysis. Furthermore, the paper proposes two methods, maximum-point-minimum-diameter (MPMD) and maximum-point-minimum-rectangle (MPMR), to reduce ALRD localization errors by gathering more beacon signals.

**Figure 9:**
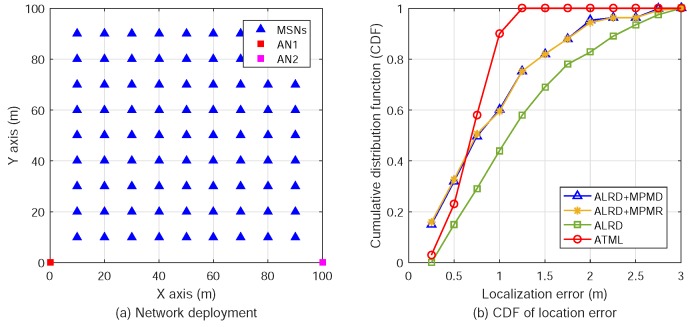
Network deployment and CDFs of the localization errors of different methods.

According to the scenario described in [28], a total of 81 MSNs and two ANs are distributed in a plane of 100×100 m^2^. The MSNs are organized in a 9×9 matrix with the intervals of 10 m. The position coordinates of the two ANs are (0,0) and (0,10) m. The deployment of the network is shown in Figure 9a. The SNR is set as 0 dB and the antenna array dimension of the ANs is 4×4. Following the same approach as in [28], we also take the average of the localization results of 10 simulation trails. The cumulative distribution functions (CDFs) of the MSN position errors by the four schemes are plotted in Figure 9b.

We can see in Figure 9b that, for very small localization error (such as smaller than 0.7 m), the ALRD-MPMD and ALRD-MPMR have higher probability (better performance) than ATML. However, for the localization error between 0.7 and 1.25 m, the probability of ATML is much larger than the other methods. The localization errors of almost all nodes are smaller than 1.25 m by ATML, but the errors of about 25% nodes are larger than 1.25 m by the other methods. Therefore, the majority of the MSNs have larger localization errors by using the three ALRD algorithms than using the ATML scheme. This indicates that ATML has a higher probability to achieve smaller localization errors than the other ALRD methods.

## 6. Testbed Development and Field Experiments

### 6.1. Prototype System and Testbed Development

**Figure 10:**
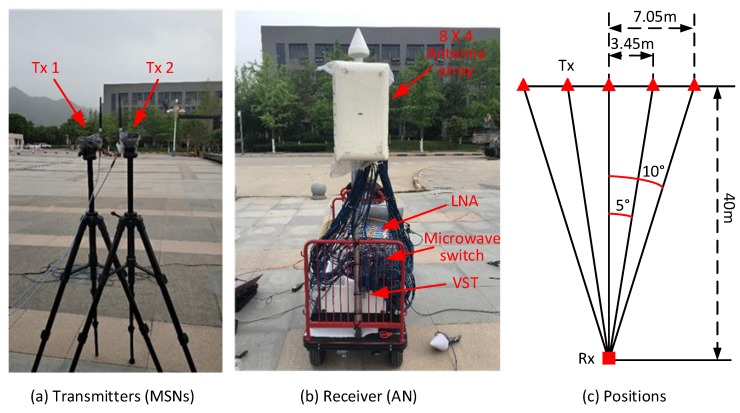
Testbed of the ATML scheme and field experiment setup.

The prototype system of the ATML scheme is implemented according to the MT-SIMO signal design and AOA ML estimation method in Section 4. The system consists of an Rx (to emulate an AN) and two Tx’s (to emulate MSNs). The system is shown in the photos in Figure 10a,b.

In each Tx, a baseband module (BBM) based on an field programmable gate array (FPGA) generates circularly the PN-sequence is the same as in the simulations in Section 5 and pre-stored in the FLASH memory. The chip rate is 20 Mcps, resulting in the chip duration of $\Delta_{c}=50$ ns and signal bandwidth of 20 MHz. The duration of the spreading sequence of $T_{u}=50\times 1023=$ 51,150 ns. The BBM also performs the waveform shaping and sampling (for digitalization) to produce the modulated zero intermediate frequency (ZIF) signal. The digital waveform samples are input into the radio frequency (RF) front-end model (FEM). The FEM first performs the frequency-up-conversion to shift the ZIF signal to the carrier of 3.5 GHz, and then drives the RF signal by an amplifier with the output power of 10 mW. Finally, the signal is transmitted by an omnidirectional dipole antenna with the maximum antenna gain of 4 dBi. The modules are controlled by an onboard microcontroller unit (MCU). All the devices are integrated circuits and suitable for practical sensor nodes due to the miniaturization.

The Rx is equipped with an 8×4 rectangular array of antenna pairs where each pair contains two $\pm 45^{\circ }$ polarized dipole antennas. The spacings between the adjacent antenna rows or columns is half a wavelength. The 64 antenna elements are connected to a microwave switch with 64 input and eight output ports. Eight antenna elements in the same polarization in one column of the array are selected by the switch and the signals received on them are output. Then, the signals pass through low noise amplifiers (LNAs) and bandpass filters, and finally are input into two vector signal transceivers (VSTs) where they are sampled at 60 MHz and stored for offline processing. The microwave switch connects eight antennas for signal capture for 320 μs and then switches to another column of eight antennas. Since there are a total of eight columns in both polarizations, the Rx takes 2.56 ms for all the 64 antenna elements to capture the signals. Although the antennas receive signals in a TDM manner, the duration is so short that the signals on all the antennas are captured almost simultaneously.

**Figure 11:**
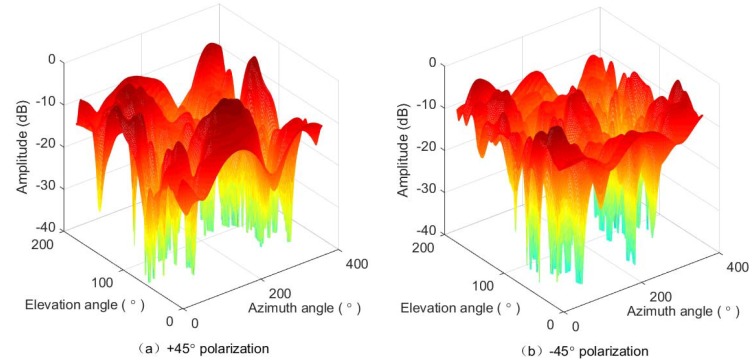
The pattern diagrams of the two cross-polarized dipoles on the (1,1) patch in the antenna array.

We have measured the 3D radiation pattern of each dipole in an anechoic chamber for $\phi \in \left[0^{\circ },360^{\circ }\right)$ and $\theta \in \left[0^{\circ },180^{\circ }\right]$ with the step of $1^{\circ }$ in the azimuth and elevation domains. For example, the pattern diagrams, $s_{i,m}\left(\phi ,\theta \right)$, of the dipoles in $+45^{\circ }$ and $-45^{\circ }$ polarizations at the position of (1,1) in the array are plotted in Figure 11. We put the whole antenna array including the back support structure into the chamber. Hence, the mutual coupling and impact among the antenna elements have been included in the measured radiation patterns. Then, the steering vectors are directly utilized in the AOA ML estimation as described in Section 4. Since the resolution of the measured pattern diagrams is $1^{\circ }$, the grain of the angle estimation error in one measurement is $1^{\circ }$. Thus, the error in one measurement is $0^{\circ },\pm 1^{\circ },\pm 2^{\circ },\cdots$. However, we can perform multiple measurement trails for one angle and take the average of multiple results. Thus, the error of final estimate may be a decimal fraction.

### 6.2. Experiment Scenario

The localization experiment was performed on a square. The location of the Rx (emulate an AN) was fixed, while the two Tx’s were mounted on two tripods and moved along a line. The experiment setup is illustrated in Figure 10c. The AOA of the LOS paths from the two Tx’s were measured when they were placed at five positions. The azimuth AOAs were $\phi_{i,k,1}=\left\{80^{\circ },85^{\circ },90^{\circ },95^{\circ },100^{\circ }\right\}$ and the elevation AOAs were always $\theta_{i,k,1}=90^{\circ }$. The threshold for the ML iteration convergence is still $\xi =10^{-3}$ degree.

### 6.3. Experiment Results

To evaluate the AOA estimation performance in the experiment, we used the relative error. For azimuth estimation, it is defined as

(20) $$e_{r,a}=\frac{\left|{\tilde{\phi }}_{i,k,1}-\phi_{i,k,1}\right|}{\phi_{i,k,1}}\times 100\%.$$

**Figure 12:**
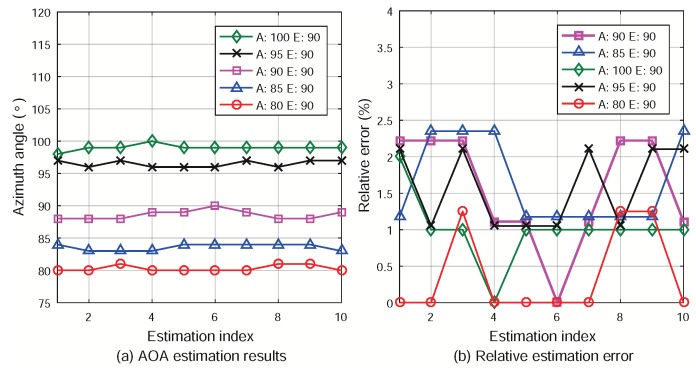
Field experiment results of AOA estimation. (A: azimuth; E: elevation).

The results and relative errors of the azimuth AOA estimation are plotted in Figure 12. We can see that the estimation errors are mostly within 2%. Therefore, the relative positions of the MSNs can be determined accurately in the experiment. The estimation errors may be introduced when the positions of the Tx’s were determined manually on the square. Adjusting the horizontal direction of the Rx antenna panel manually may also affect the accuracy of the AOA estimation.

As indicated by the simulation results, AOA estimation error mainly depends on the antenna array dimension and the number of MSNs. The antenna array used in the field experiment had the horizontal dimension of four antennas. In the simulation results, when the number of MSNs equals 10 and the antenna array dimensions is 4×4, the angle estimation error is about $0.5^{\circ }$. The angle estimation error in the measurement results is mostly below $2^{\circ }$. The simulation and experimental results are in the same order, and the latter is slightly larger than the former. This may be caused by, as mentioned earlier, the manual setup of the MSN positions and the attitude of the antenna panel. Therefore, the field experimental results agree with the simulation results reasonably.

The computational complex and energy consumption of the ATML scheme are analyzed as following.

1. The joint estimation of azimuth and elevation AOAs (${\tilde{\phi }}_{i,k,1}$ and ${\tilde{\theta }}_{i,k,1}$) brings great difficulty to ML estimation. In ATML, the two AOAs are estimated separately and iteratively. Thus, the multi-parameter ML estimation is divided into single-parameter ML estimation, which greatly reduces the algorithm complexity.
2. The simulation and experimental results show that the iterative ML algorithm can achieve convergence quickly (e.g., within three steps) and achieve high estimation accuracy. Therefore, the computational load and resources requirements are low.
3. An MSN can broadcast a spreading code with very low transmission power (e.g., 10 mW in the field experiment). By taking advantage of spread spectrum gain and antenna array gain, ATML can ensure a high SINR to achieve high accuracy in AOA estimation and 3D multi-target localization. Hence, ATML has high energy efficiency for MSNs.

## 7. Conclusions

In this paper, an ATML approach for locating the 3D positions of multiple MSNs simultaneously in a SIWSN is proposed. The MT-SIMO signal transmission scheme is designed by which two ANs capture the spread spectrum signals broadcast by MSNs using antenna arrays. The 2D AOAs of the LOS paths from every MSN to both ANs are estimated by using a simple iterative MLE based on the LPRVs. Based on the estimated AOAs and the skew line theory, the positions of multiple MSNs are determined. A testbed has been implemented for ATML. The simulation and field experiment results validate the effectiveness and feasibility of the proposed ATML in practice. It is shown that the ATML can estimate the positions of multiple MSNs simultaneously in a large range of SNR and with small antenna array dimensions. By exploiting the spread spectrum gain and the array gain, the ATML can achieve high accuracy and efficiency in multi-target localization. How to further improve the positioning accuracy, for example, by using the Kalman filtering and multi-domain signal fusion, will be investigated in our future works. To enhance the security of the electronic systems on MSNs for industrial applications is also an important issue to study [29,30]. 

## Figures and Tables

**Table 1:** Propagation path parameters.

Parameters	Values	Parameters	Values
number of MSNs (*K*)	10∼70	number of paths (*L*)	5
antenna array size (*M*)	2 × 2∼8 × 8	received SNR	−30∼30 (dB)
PN-sequence length (*N*)	1023 chips	chip rate	20 Mcps
convergence threshold (ξ)	$10^{-3}$ degree	signal bandwidth	20 MHz

**Table 2:** Propagation path parameters.

MSN	$\tau_{l}$ (Chips)	$|\alpha_{l}{|}^{2}$	$\phi_{l}\;{(}^{\circ })$	$\theta_{l}\;{(}^{\circ })$	$\nu_{l}$ (KHz)
$M_{1}$	1	20	30	40	1
$M_{2}$	2	18	45	60	5
$M_{3}$	5	14	85	75	2
$M_{4}$	8	12	95	100	3
$M_{5}$	12	8	120	130	8
